# The Fight against the Carcinogenic Epstein-Barr Virus: Gut Microbiota, Natural Medicines, and Beyond

**DOI:** 10.3390/ijms24021716

**Published:** 2023-01-15

**Authors:** Radwa A. Eladwy, Hang Thi Vu, Ravi Shah, Chun Guang Li, Dennis Chang, Deep Jyoti Bhuyan

**Affiliations:** 1 NICM Health Research Institute, Western Sydney University, Penrith, NSW 2751, Australia; 2 Faculty of Food Science and Technology, Vietnam National University of Agriculture, Trau Quy, Gia Lam, Hanoi 100000, Vietnam

**Keywords:** oncogenic viruses, Epstein-Barr virus, gut microbiota, natural products, short-chain fatty acids, gut metabolites

## Abstract

Despite recent advances in oncology, cancer has remained an enormous global health burden, accounting for about 10 million deaths in 2020. A third of the cancer cases in developing counties are caused by microbial infections such as human papillomavirus (HPV), Epstein-Barr Virus (EBV), and hepatitis B and C viruses. EBV, a member of the human gamma herpesvirus family, is a double-stranded DNA virus and the primary cause of infectious mononucleosis. Most EBV infections cause no long-term complications. However, it was reported that EBV infection is responsible for around 200,000 malignancies worldwide every year. Currently, there are no vaccines or antiviral drugs for the prophylaxis or treatment of EBV infection. Recently, the gut microbiota has been investigated for its pivotal roles in pathogen protection and regulating metabolic, endocrine, and immune functions. Several studies have investigated the efficacy of antiviral agents, gut microbial metabolites, and natural products against EBV infection. In this review, we aim to summarise and analyse the reported molecular mechanistic and clinical studies on the activities of gut microbial metabolites and natural medicines against carcinogenic viruses, with a particular emphasis on EBV. Gut microbial metabolites such as short-chain fatty acids were reported to activate the EBV lytic cycle, while bacteriocins, produced by *Enterococcus durans* strains, have shown antiviral properties. Furthermore, several natural products and dietary bioactive compounds, such as curcumin, epigallocatechin gallate, resveratrol, moronic acid, and andrographolide, have shown antiviral activity against EBV. In this review, we proposed several exciting future directions for research on carcinogenic viruses.

## 1. Introduction

Cancer has remained a serious concern and is the second leading cause of death worldwide [1]. The transformation of normal cells into neoplasm as a result of external stimuli, including physical carcinogens (such as UV), chemical carcinogens (such as asbestos), and biological carcinogens (such as viral and bacterial infections), can lead to various cancers [2]. The early link between cancer and viral infections was studied in chickens in 1910 by the Nobel Laureate Peyton Rous [3]. Since then, several viruses with their oncogenic mechanisms have been discovered. To date, eight oncogenic viruses (both RNA and DNA viruses) have been identified, which can induce cancer by different mechanisms [4]. 

Oncogenic viral infections contribute to about 15% to 20% of total cancer in humans [5]. These known human oncoviruses include the hepatitis B virus (HBV), hepatitis C virus (HCB), Epstein-Bar virus (EBV), human T-lymphotropic virus 1 (HTLV-1), Kaposi sarcoma-associated herpesvirus (KSHV; also known as human herpesvirus 8), human papillomaviruses (HPV), Merkel cell polyomavirus (MCPyV), and human immunodeficiency virus (HIV) [6]. Oncoviruses are infectious agents that cause prolonged, persistent infections that can induce the incidence of tumours [7,8,9]. For instance, HIV, rather than causing cancer directly, induces carcinogenesis of other viruses, including EBV and KSHV [6]. According to the classic carcinogenesis theory, initiators and promoters act on cells in multiple steps or ‘hits,’ which sets the transformation process, invasion, and metastasis [10,11]. The accumulation of these multiple hits is required for full cancer formation [12] and this whole process may take several years [12]. ‘Hits’ are considered genetic and defined by amplifications in oncogenes, mutations & rearrangement, or changes in tumour suppressor genes [13].

**Figure 1:**
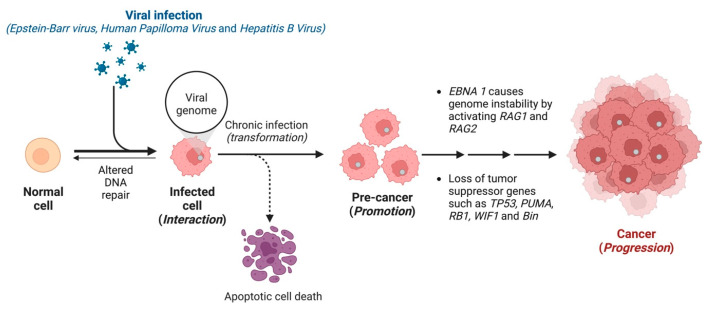
Transformation of normal cells into cancer cells by virus-mediated carcinogenesis.

The transformation of a normal cell into a cancer cell by virus-mediated carcinogenesis involves initiation, promotion, and progression (Figure 1) [14]. An interaction between a carcinogen and the host DNA is initiation, whereas promotion is when cell proliferation occurs (which usually takes a few months to years). The final step is the spread of the tumour, known as progression. Human oncoviruses display direct and indirect cell transformation mechanisms (viral carcinogenesis) (Figure 1) [15]. The virus encodes genes that activate growth and enhances apoptosis resistance for direct carcinogenesis, therefore, modifying the DNA repair mechanism [4,16]. During viral oncogenesis, tumour suppressors like p53 and pRb are inactivated until the DNA repair process is restored, otherwise, cell death is activated (Figure 1) [16]. Our genome also tends to acquire mutation, which is increased during viral infections. For example, viral antigen EBNA-1 can cause genome instability by activating *RAG1* and *RAG2* (Figure 1) [3].

Oncogenic viruses maintain telomere length by interfering with telomerase expression leading to uncontrollable division [17]. Viral antigens like Kaposi’s sarcoma-associated herpesvirus latency-associated nuclear antigen (LANA), HPV E6, HBV HBx, and latent membrane protein 1 of the Epstein-Barr virus have been found to enhance telomerase activity [18]. MicroRNAs have been found to inhibit the translation of mRNA [19]. These microRNAs target cellular tumour suppressor genes like *WIF1, Bin*, and *PUMA* and inhibit cell death [20]. For indirect carcinogenesis, viruses do not live in the cancer cells but act through mechanisms such as chronic inflammation, immune suppression, and oncomodulation, leading to carcinogenic mutations in the host cells [21,22,23].

Standard antivirals, including acyclovir and ganciclovir, can prevent EBV and its lytic replication but have not received Food and Drug Administration (FDA) approvals [24]. Additionally, the use of acyclovir and ganciclovir is linked to thrombocytopenia, liver and kidney damage, and gastrointestinal dysfunction [25]. Therefore, discovering novel, potent, and safe antiviral agents is crucial. An analysis of medicinal chemistry annual reports from 1984 to 1995 indicated that the majority of the synthetic agents approved by the FDA are derived from medicinal plants [26], however, critical and comprehensive reviews on the activity of these natural bioactive molecules against oncogenic viruses are limited.

Gut microbiota is the collection of eukarya, archaea, and bacteria colonising the host’s gastrointestinal tract (GIT). It coevolved over thousands of years through symbiosis with the host in a complex and mutually beneficial relationship. Studies on gut microbiota have just started in the last 20 years, despite its pivotal role in human health and disease pathogenesis. Our group has recently reviewed the fundamental role of gut microbiota and its metabolites in the development and treatment of cancer [27,28]. In the current review, we summarise and discuss the activity of gut microbial metabolites and natural products against carcinogenic viruses, emphasising EBV, and their implementation in anticancer drug development in the future.

## 2. Epstein–Barr Virus

**Figure 2:**
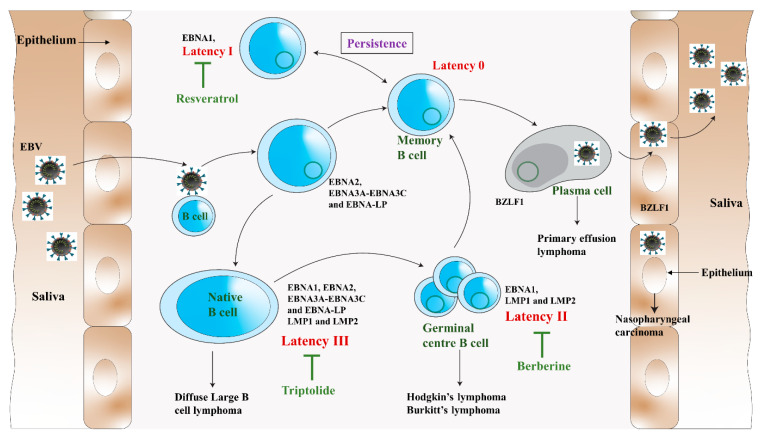
The impact of resveratrol, triptolide, and berberine on different phases of the EBV life cycle (modified from Münz, 2019 [33])**.** The virus passes from saliva to the B lymphocytes crossing the oropharyngeal epithelium. Here, the virus causes the B cell to multiply and outspread through the B cell compartment. The infected B cells may differentiate into latency 0 or total latency III transformation. The latency III phase is highly immunogenic and *EBNA1, EBNA2, EBNA-LP, EBNA3 (A, B, C)*, and *LMP (1,2)* are expressed. To remain immune silent, the virus switches from latency III to latency II in which *EBNA1, LMP1,* and *LMP2* are expressed, and then to latency 0. For the persistence of viral infection, EBV drives latency 0 to latency I, where *EBNA1* is only expressed to allow the EBV episome to be dissociated and maintained in dividing B cells. These B cells will randomly go for the lytic cycle and lyse to produce and release the virions back to the saliva to infect more host B lymphocytes. The germinal centre differentiation pathways provide premalignant precursors for Hodgkin’s lymphoma and non-Hodgkin’s lymphomas, including diffuse large B-cell lymphoma and Burkitt’s lymphoma. The lytic cycle primarily occurs in the plasma cells.

EBV, formally called the human gamma herpesvirus 4, is one of the most common viruses in humans [29]. It infects more than 90% of the population worldwide [30]. Primary infection during childhood is generally asymptomatic. However, the virus can cause infectious mononucleosis in 35–50% of the cases when infection occurs later in life [24]. EBV infects 90–95% of all adults globally and causes approximately 1% of all cancers and is implicated in around 2% of all cancer-related deaths [31]. EBV is associated with several human malignancies, including Burkitt lymphoma, Hodgkin disease, nasopharyngeal carcinoma (NPC), gastric cancer, T/NK lymphoma (nasal natural killer/T-cell lymphoma), AIDS, and transplantation-associated lymphomas [30]. The virus has two cycle stages, the latent and lytic cycles [24]. In latency, the virus expresses only a limited number of genes, including *EBNA 1-6*, *LMP1*, and *LMP2* [24]. In the lytic cycle, EBV expresses two transcription factors, Rta (R transactivator, the product of *BZLF1* gene) and Zta (Z EB replication activator, the product of *BZLF1*). These two proteins trigger lytic genes, which encode diffused early antigen (EA-D) and DNA polymerase (Figure 2). Positive regulators of *BZLF1* and *BRLF1* genes can trigger the shift from the latency to the lytic cycles of EBV. Activators of *BZLF1* and *BRLF1* genes in B cells include physiological stimuli, B-cell receptor engagement, DNA damage, TGF-β, hypoxia, and chemical agents [32].

After DNA replication, late genes are expressed, followed by the production of virus capsid antigens and membrane protein, and then they produce the mature virion [24]. The lytic infection was shown to contribute to lymphoproliferative disease [24]. Therefore, the antiviral agent that blocks the viral lytic cycle, such as resveratrol, triptolide, and berberine, can provide potential treatment or prevention strategies for EBV-associated diseases (Figure 2).

EBV latency in resting and proliferating cells is divided into types III, II, I, and 0, depending on the viral gene expression pattern [33]. It was reported that EBV induces cancers of epithelial and lymphoid origin by the transformation of quiescent B cells into lymphoblastoid cell lines (LCLs) (Figure 2) [34]. The virus life cycle is confined chiefly to the B cells, establishing premalignant latent gene expression patterns. When EBV infects a naïve B cell, it expresses the latency III or growth program [35]. In this pattern, the virus expresses *EBNA 1-6, LMP1, LMP2A*, and *LMP2B* with a complete oncogenic component of nuclear proteins EBNA2, 3A, 3B, 3C, LP, and the EBV microRNAs. The infected B cell then becomes proliferating B-cell which helps in EBV episomes replication. Notch signalling is dysregulated in this gene expression pattern, leading to nonviral lymphoid malignancies. Expression of *c-myc*, a cellular proto-oncogene, is dysregulated by upregulating cyclin Ds and E and downregulating CDK2 inhibitors like p21^CIP1^ and p27^KIP1^. Latency III has a free pattern of gene expression resulting in the production of oncogenic proteins and eventually can cause cancer, especially in immunocompromised patients [36]. In immunosuppressed and AIDS patients, AIDS-related-non-Hodgkin lymphoma and post-transplantation lymphoproliferative disease latency III expressions are common [37].

Latency III is the most immunogenic phase of the EBV life cycle. Therefore, the cells express a pattern shift to a default program or latency II to remain partially immune silent (Figure 2). *EBNA1* and two oncogenes, *LMP1* and *LMP2A*, are expressed in latency III [35]. These two oncogenes are essential for survival and B cell proliferation [38]. *LMP1* activates NF-_k_B, a key transcriptional factor in viral and nonviral lymphomagenesis which upregulates antiapoptotic molecules like A20 and Bcl2 in B cells to promote cell proliferation and malignancy [35]. *LMP1* inhibits DNA damage response and enhances telomerase activity via JNK activation to promote genetic instability [39]. Likewise, *LMP2A* helps in B cell differentiation, survival, and cell growth when the Ig receptor expression is unavailable through PI3K-AKT pathway activation [40]. AIDS-associated lymphomas and post-transplant lymphoproliferative disorders are common during latency III, while diseases like Hodgkin’s disease and nasopharyngeal carcinoma are associated, during latency II [36].

Burkitt’s lymphoma is common in latency I. The infected cell can switch to immunologically silent latency programs. In this expression pattern, *EBNA1* is only expressed to allow the EBV episome to be disassociated and maintained in dividing B cells [35,41] (Figure 2). EBV replicates either by B cell proliferation or by lytic replication. In a cell culture setting, some chemicals like sodium butyrate and 12-*O*-tertradecanoylphorbol (TPA) can induce the EBV lytic cycle [42]. It suggests that host cell signal transduction and epigenetic regulation play an essential role in switching the virus between latency and lytic replication. When lytic replication occurs, the virus expresses almost all the genes present in its genome [33]. For instance, genes like *BALF1, BCRF1*, and *BARF1* are expressed during the lytic cycle, with oncogenic consequences [36]. The early lytic EBV replication creates a tumour microenvironment responsible for the observed increased tumorigenesis [33]. Also, more TNF, CCL5, and IL-10 are produced by LCLs with an increased level of spontaneous lytic EBV reactivation, which recruits immunosuppressive myeloid cells (such as TNFα-CCL5-macrophages) and inhibits the immune control by cytotoxic lymphocytes. From a preclinical in vivo setting, EBV-associated lymphomagenesis increases with lytic EBV replication through the formation of a tumour microenvironment, and suppression of this lytic replication might be a useful strategy for the treatment of EBV-induced malignancies.

Several antiviral compounds designed for other herpesviruses have been tested on EBV. These antiviral compounds can be divided into two major categories, (a) those that interfere with the virus-encoded enzymes, and (b) the ones that interfere with the cellular processes required for virus production [24]. The former includes nucleoside analogues and non-nucleoside analogues. The other category comprises drugs targeting cellular protein. Although several antivirals were proven to inhibit EBV replication in vitro, they had limited success in clinical trials [24]. The current literature reveals that no antiviral drug has been approved to treat EBV infections [24,25,43].

## 3. Gut Microbiota

**Figure 3:**
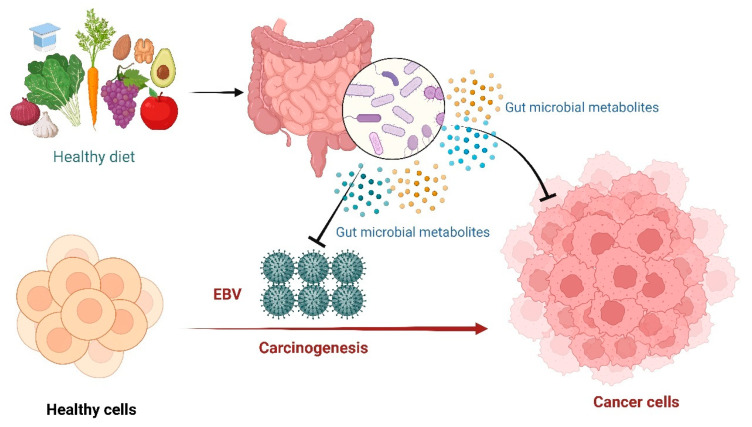
Epstein-Barr Virus (EBV) medicated carcinogenesis and the potential protecting role of gut microbiota through the production of metabolites.

Microbiota refers to a vast number of interacting bacteria, fungi, eukaryotic viruses, archaea, and bacteriophages coexisting with the host for potential mutual benefit [44]. In the last two decades, extensive research has been done to explore the nature and therapeutic potential of gut microbiota [45]. Emerging evidence has elucidated the role of gut microbiota in protecting from pathogens, maintaining metabolic, endocrine, and immune functions, and modifying drug action and metabolism (Figure 3) [45]. Gut microbial metabolites include short-chain fatty acids, inosine, and bacteriocins, which have shown a broad spectrum of biological activities in previous studies including anticancer and immunomodulatory functions [27,46,47]. 

### 3.1. Probiotics and EBV

Probiotics are living organisms that provide beneficial effects to the host when consumed in sufficient amounts. *Lactobacilli* and *Bifidobacteria* are the most common probiotics [48]. Probiotics have been used as antimicrobials and for the prevention and management of different allergies [48]. For instance, supplementation of *Lactobacilli casei* (strain Shirotais) (6.5 × 10^9^ CFU per day) to endurance athletes for 20 weeks reduced the plasma levels of EBV and CMV (cytomegalovirus) antibodies, presumably by improving the host immunity [49]. 

### 3.2. Short-Chain Fatty Acids (SCFAs)

**Figure 4:**
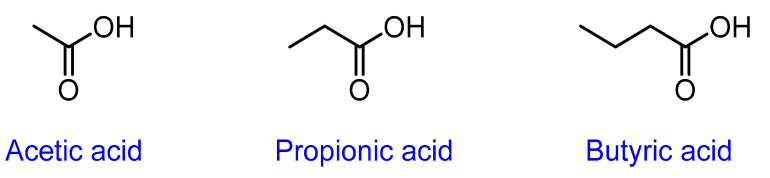
Chemical structure of short-chain fatty acids.

SCFAs are formed principally by fermentation of undigested starch and nonstarch polysaccharides in the large bowel [50]. These are small monocarboxylic acids comprised mainly of acetic (or acetate), propionic (or propionate), and butyric (or butyrate) acids (Figure 4) [50].

SCFAs are metabolised by the colonocytes and the unmetabolized fractions are transported into the portal circulation to be used as an energy source for the hepatocytes, except for acetate which is barely oxidized in the liver [51]. As a result, only a small amount of SCFAs, originating from the colon, reaches the systemic circulation, so their faecal concentration has been used as a proxy for SCFAs production in the colon [45]. Many studies have shown the beneficial role of SCFAs in different diseases, including diabetes, cancer, and hypertension. Table 1 summarises the potential therapeutic use of SCFAs and their proposed mechanisms of action. Several reports demonstrated the protective role of SCFAs against infections primarily attributed either to their direct antivirulence effect or indirect effect on the host immune system [52]. Recently, SCFAs showed antiviral effects against respiratory syncytial virus bronchiolitis and, interestingly, it is also reported that SCAFs can activate the EBV lytic cycle [52,53]. In particular, propionate and butyrate have been found to activate the EBV lytic cycle. They have also been reported as general inhibitors of class I and class II histone deacetylases in different cell types [53].

### 3.3. Inosine

**Figure 5:**
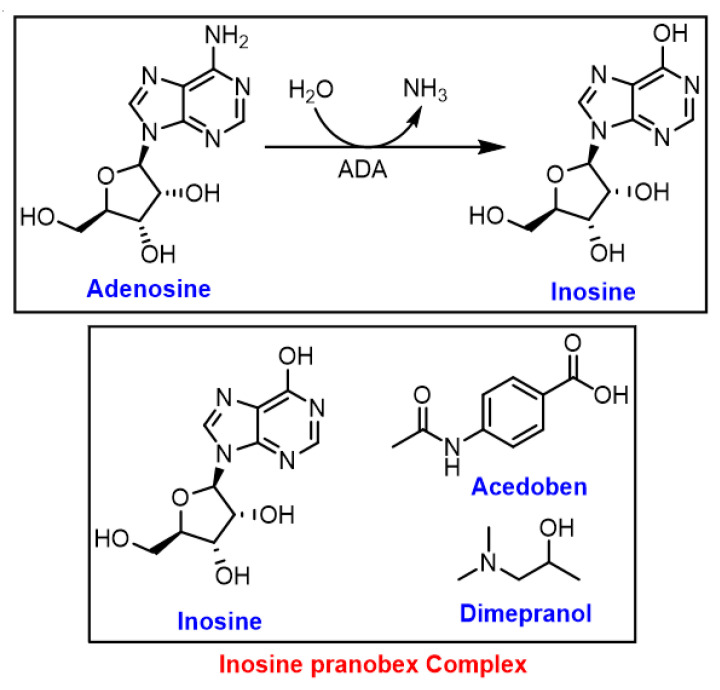
Formation of inosine by specific RNA deaminase; ADA: adenosine deaminases.

Inosine, the key intermediate in purine metabolism, is produced via the deamination of adenosine by specific RNA deaminases (Figure 5) [57]. In living organisms, inosine is an abundant nucleic acid base, and it serves as a crucial intermediate in purine metabolism [57]. In a recent in vivo study, the enhanced immunotherapy response by intestinal *Bifidobacterium pseudolongum* was modulated mainly by inosine metabolite and it was dependent on T cell expression of the adenosine A2A receptor [58]. 

Isoprinosine (IP), known as inosine pranobex or methisoprinol, is a 1:3 complex of inosine and pranobex, respectively (Figure 5) [46]. The latter is a mixture of dimethyl amino isopropanol (dimepranol) and p-acetamidobenzoate (acedoben) (Figure 5) [46]. Since 1971, IP has been widely used against viral infections, including the herpes simplex virus (HSV), HPV, HIV, cytomegalovirus (CMV), influenza, and acute respiratory infections, as well as EBV infections, primarily due to its immunomodulatory effect and safety profile. The antiviral properties of IP, when given after the onset of virus diseases, is due to enhancing the natural immune response of lymphocytes [46].

Table 2 summarises the key in vivo studies of isoprinosine against different viral infections. The mechanisms of action of IP as an immunomodulator in viral infections have been proposed to be through increasing the levels of proinflammatory cytokines, such as IL-2 and INF-c in mitogen- or antigen-activated cells, which initiates T-lymphocyte differentiation resulting in induced lymphoproliferative action [59,60]. INF-c, in combination with the direct effect of IP, decreases the production of anti-inflammatory cytokines, including IL-10 suggesting the potential immunomodulatory effect of IP on innate and adaptive immunity. IP was also reported to increase the natural killer (NK) cells population and activity and potentiate phagocytosis and macrophage chemotaxis [61,62,63].

The in vivo antiviral activity of IP was evaluated on murine gammaherpesvirus 68 (MHV68), which is a natural pathogen of mice, and used as a model for EBV infection. After 2 weeks of treatment, IP resulted in elevated levels of virus-neutralizing antibodies, leukocytes, and neutrophils. However, the antiviral effect was time-limited and disappeared after 120–150 days. The incidence of tumour formation was 7.5% in the MHV-infected group after treatment with IP, compared to 17.5% in the untreated group. This study showed that IP administration should be repeated in chronic EBV infections [64].

**Table 2:** Summary of key studies investigating the efficacy of isoprinosine (IP) in different viral infections.

Infection	Type of Study	Main Findings	References
Epstein-Barr virus	In vitro	The protective immunomodulating effect of IP was investigated on EBV-transformed B lymphoid cells. IP was found to be an immunomodulating agent with an ability to enhance the response of sensitized peripheral blood mononuclear cells (PBL) to EBV-antigens.	[65]
Acute respiratory viral infections	Clinical: Randomised, double-blind, Phase 4 study (n = 231) with placebo (n = 232)	Faster improvement in subjects in the IP group versus those in the placebo group.	[66]
SARS-CoV-2 (COVID-19)	Clinical: The study was conducted during June-September 2020 in three nursing homes (in the Czech Republic) with 301 residents, 156 of whom (51.8%) tested positive for the SARS-CoV-2 virus (PCR test).	Demonstrated the positive effect of IP against COVID-19. The case-fatality rate in these three nursing homes at the end of the pandemic’s first wave was lower than similar nursing homes in the Czech Republic.	[67]

### 3.4. Bacteriocins

Bacteriocins are ribosomally-synthesized small amphiphilic peptides produced by archaea and bacteria and extracellularly transported via an ATP-binding cassette transporter [47]. Several bacteriocins produced by the lactic acid bacteria (LAB) display bactericidal or bacteriostatic effects on similar or closely related bacterial strains [68]. They are fundamentally classified into two classes, class I lantibiotics and class II nonlantibiotics [47]. Lantibiotics, are gene-encoded peptides that are nontoxic to humans and have versatile applications as antibiotics, biopreservatives, or bactericidal agents in cosmetics [47]. They act by forming pores in the cell membrane resulting in a decrease in the intracellular pH and efflux of small metabolites [47]. Class I (lantibiotics) bacteriocins include the single peptides nisin, mersacidin, and lacticin, while class II (non-lantibiotics) include pediocin, lactacin, and lactococcin. Although a precise mechanism of action for every bacteriocin is still unclear, binding to lipid II, the main transporter of peptidoglycans from the cytoplasm to the cell wall, is reported in different studies [47]. Several reports documented the antibacterial properties of bacteriocin. However, very few reports to date have displayed their potential antiviral effect [69]. In particular, previous studies demonstrated the antiviral properties of bacteriocins against different viruses including the oncogenic viruses HSV-1 and HSV-2 [70,71,72,73].

## 4. Natural Products as Antiviral Agents against EBV

Several oncogenic viruses have been identified since the association between viruses and cancer was discovered in 1910 [3]. Even though the vaccines against HBV and HPV have been highly successful, more preventative and therapeutic options are necessary for other oncogenic viruses. Recent research has focused on the recovery of antivirals from medicinal plants and foods, such as curcumin, andrographolide, resveratrol, epigallocatechin gallate (EGCG), and moronic acid (Table 3) [74,75,76]. However, more research is necessary to discover novel, potent, and safe natural antiviral agents against oncoviruses. Bioactive molecules from various natural sources, such as bryostatins, didemnin, cephalostatin from marine organisms, silymarin, stilbenes, vincristine from plants, rhizoxin, mitomycin, rapamycin from microorganisms, curcumin, allicin, and β-carotene from fruits, vegetables, and herbs have been analysed in vitro for their potential activity against oncogenic viruses [4,77,78,79]. However, their molecular mechanisms of action have not been investigated adequately, and further studies are crucial to understand their efficacy in living organisms through in vivo studies. Few natural medicine products including berberine, manassantin B, grifolin, and triptolide have been tested both in vivo and in vitro in relation to the EBV antiviral and anticancer mechanisms) [80]. The summary of plants and natural bioactive compounds with antiviral effects against EBV is presented in Table 3. The following section highlights the most interesting findings.

*Andrographis paniculata* is a medicinal plant commonly used in traditional medicine in Asia. The herb has a bitter taste and is used to treat the common cold, colic pain, liver disorders, and bowel issues [75]. *A. paniculata* was reported with several biological properties such as immunoregulatory, anti-HIV, and antibacterial activities in previous studies [75]. Recent research showed the antiviral effect of the ethanolic extract of *A. paniculata* against EBV [75]. The mechanism of inhibition was reported to occur via blocking the transcription of the immediate–early genes that encode lytic proteins Rta and Zta. *Andrographolide* was the compound of interest in *A. paniculata* which effectively inhibited EBV at 5 µg/mL [75]. Furthermore, the compound was found to be non-toxic to the P3HR1 cells at that concentration, indicating its potential anti-EBV effect.

*Polygonum cuspidatum* is another popular medicinal plant in Asia, which has been used as a hepatoprotective and cholagogic traditional medicine in China, Japan, and Korea [81]. The ethanolic extract of *P. cuspidatum* was reported to inhibit the transcription of EBV immediate early genes, and the expression of lytic proteins Rta, Zta, and EA-D [82,83]. The extract also inhibited the expression of latent membrane protein one triggering the EBV-positive cells toward apoptosis [82]. In addition, the ethyl acetate subfraction from ethanolic extract displayed a more potent EBV inhibitory effect [84]. The subfraction blocked the transcription of immediate early genes, reduced DNA replication, and hindered the production of virus particles [84]. Furthermore, the primary active components in *P. cuspidatum* were reported to be resveratrol and emodin. The effective concentration of emodin required to inhibit the expression of immediate–early protein by 50% (EC_50_) obtained from flow cytometry was 4.83 μg/mL (17.87 μM) and its EC_50_ value to reduce DNA replication was 1.2 μg/mL [84]. These values were substantially lower than those of resveratrol, EGCG, and andrographolide, suggesting that emodin effectively inhibited the EBV lytic cycle and can be a potential lead for drug development for EBV-related diseases [84].

Diterpenoids are common secondary metabolites found in plants such as *Scutellaria barbata* and *Euphorbia milii. E. milii* has been used in Chinese medicine for detoxifying purposes for centuries [85]. This group of compounds exhibits a wide range of biological activities including antimicrobial, anti-inflammatory, antitumour, and antiviral effects. Neo-clerodane diterpenoids from *S. barbata* showed inhibition toward the EBV lytic cycle [86]. Similarly, diterpenoids in *E. milii* were reported to have potential antiviral activity. In particular, the acetone extract of *E. milii* inhibited the EBV lytic cycle [87]. Thirteen new entrosane-type diterpenoids (1–13) were isolated from the *E. milii* extract and evaluated against EBV. Among those, one derivative showed the most potent inhibitory activity with an EC_50_ value of 5.4 μM compared to the positive control (+)-rutamarin (EC_50_ = 5.4 μM) [87].

**Figure 6:**
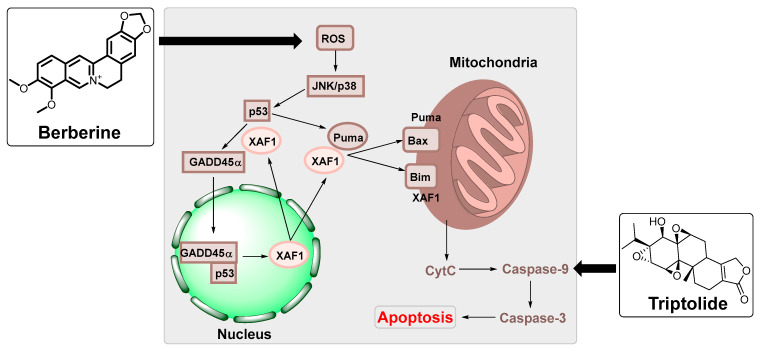
A diagrammatic representation of the intracellular signalling mechanism during berberine-induced apoptosis in EBV-positive B lymphoma cells (modified from Park et al. [89]). In this process, intracellular reactive oxygen species (ROS) production is stimulated upon berberine treatment, leading to the activation of various molecules, ultimately resulting in caspase-dependant apoptosis of EBV-positive B lymphoma cells [89]. Triptolide was reported to induce apoptosis via caspase-9 dependant cell death mediated through the mitochondrial pathway [95].

Berberine is a natural compound found in the rhizome, roots, and bark of medicinal plants such as barberry (*Berberis vulgaris*) and huanglian (*Coptidis rhizome*) (Figure 6) [88]. Several studies showed the potential anti-inflammatory and chemo-preventative effects of berberine [88]. It has been illustrated that berberine can inhibit cell proliferation, cell cycle arrest, and apoptosis of EBV-associated NPC cells [89]. Berberine targeted *EBNA1* transcription and downregulated its expression, inhibited the p-STAT3 expression, and decreased EBV virions production [90]. The in vivo results of a nontoxic dose of berberine showed a decrease in tumour growth of the EBV-associated NPC [91]. In addition, berberine-induced apoptosis illustrated the activation of XAF1 and GADD45a (Figure 6) [89]. Berberine increased the cellular reactive oxygen species levels and upregulated p53 via activation of JNK and p38-MAPK pathways. Then p53 translocated GADD45α (growth arrest and DNA damage-inducible alpha) protein into the nucleus and the XAF1 (X-linked inhibitor of apoptosis 1) protein into the cytosol. Furthermore, p53 upregulated PUMA, a proapoptotic protein that rapidly induced apoptosis through a bax- and mitochondrial-dependent pathway (Figure 6) [89].

Triptolide produced by the thunder god vine (*Tripterygium wilfordii*) was reported to have anti-inflammatory, immunosuppressive, and anticancer activities [92]. Studies have demonstrated that cell proliferation of EBV-positive B lymphoma cells can be inhibited by triptolide via downregulation of *LMP1* transcriptional expression [93]. Triptolide targeted the *LMP1* promoter in type III infection cells, downregulating the *LMP1* mRNA [93]. In comparison, other studies showed that triptolide inhibits the *EBNA1* expression by increasing the sensitivity of mitochondrial apoptosis in NPC [94]. 

**Table 3:** In vitro and in vivo biological activity of some natural products against Epstein-Barr Virus.

Compound	Source	Cell Line/Animal	Mode of Action	References
Berberine	*Berberis vulgaris*	HK1 and HONE1 nasopharyngeal carcinoma cells and NOD/SCID mice	Suppressed the expression of *EBNA1*.	[88]
		C666-1, HONE1, HK1 and NP460 nasopharyngeal carcinoma cells and nude mice	STAT3 activation was inhibited in the nasopharyngeal carcinoma cells.	[90]
		CNE2 nasopharyngeal carcinoma cells and BALB/C-NU male mice	Regulated the expression of key proteins of the MAPK/ERK pathway.	[91]
		IM-9 multiple myeloma cells	Upregulated XAF1 and GADD45a expression by MAPK and functional p53.	[89]
Resveratrol	*Polygonum cuspidatum*	Raji Burkitt lymphoma cells and female mice	Activation of TPA-induced EBV-early antigen was suppressed.	[76]
		P3HR-1 Burkitt lymphoma cells	Decreased the growth and development of the virus.	[96]
		Akata and Raji Burkitt lymphoma cells	Decreased protein synthesis and lowered reactive oxygen species (ROS) levels. Redox-sensitive transcription factors AP-1 and NF-kB were suppressed.	[97]
		Akata and B95-8 Burkitt lymphoma cells	Antiapoptotic proteins Mcl-1 and survivin were downregulated.	[98]
Apigenin	*Punica granatum*	NA and HA nasopharyngeal carcinoma and P3HR1 Burkitt lymphoma cells	Suppressed the promoter activities of two viral *IE* genes and decreased the viral reactivation	[99]
(+)-Rutamarin	*Rutu graveolens*	P3HR-1 Burkitt lymphoma cells	Exhibited anti-EBV lytic DNA replication.	[100]
Wogonin	*Scutellaria baicalensis*	Raji Burkitt lymphoma cells and four-week-old male BALB/c nude mice.	The expression of NF-*k*B was downregulated through LMP1/miR155/NF-kB/PU.1 pathway.	[101]
Epigallocatechin gallate	*Camellia sinensis*	P3HR-1 Burkitt lymphoma cells	Inhibited the EBV lytic proteins expression.	[102]
		CNE1-LMP1 nasopharyngeal carcinoma and B95-8 Burkitt lymphoma cells	The MEK/ERK1/2 and PI3K/AKT signalling were suppressed.	[103]
			Downregulated *LMP1*.	[104]
Glycyrrhizin	*Glycyrrhiza glabra*	HEK (Human embryonic kidney) cells	Inhibited EBV infection. Targeted the first step of the SUMO (small ubiquitin-like modifier)ylation process resulting in limited cell growth and apoptosis.	[105,106]
Triptolide	*Tripterygium wilfordii*	HONE1/Akata, C666-1 nasopharyngeal carcinoma and P3HR-1 cells Burkitt lymphoma cells	Reduced the expression of *LMP1* in EBV-positive B lymphocytes.	[93]
		HEK and B95-8 Burkitt lymphoma cells, HeLa (cervical cancer cells and BALB/c male mice	Increased the mitochondrial apoptosis sensitivity in nasopharyngeal carcinoma cells.	[94]
		HEK, B95-8 and P3HR-1 Burkitt lymphoma cells	Targeted and downregulated the translation factors SP1 and c-Myc.	[107]
Phytol	*Lindernia crustacea*	P3HR-1 Burkitt lymphoma cells	RTA expression was inhibited.	[108]
Aloe-emodin	*Lindernia crustacea*		EBV lytic cycle was inhibited.	[108]
Cis/trans-martynoside & Cis/trans-isomartynoside	*Lindernia crustacea*		EBV lytic cycle was affected.	[108]
Emodin	*Polygonum cuspidatum*	P3HR-1 Burkitt lymphoma cells	EBV lytic cycle was affected. Inhibited the transcription of EBV immediate early genes, the expression of EBV lytic proteins, and reduced EBV DNA replication.	[84]
		NA, HA, HONE-1 and TW01 nasopharyngeal carcinoma cells	EBV reactivation and nasopharyngeal carcinoma recurrence were decreased.	[109]
(+)-Hyperjaponicol B & Hyperjaponicol D	*Hypericum japonicum*	B95-8 Burkitt lymphoma cells	Suppressed EBV DNA replication.	[110]
Hyperjaponicol H	*Hypericum japonicum*	B95-8 Burkitt lymphoma cells	Moderately inhibited EBV lytic DNA replication.	[111]
Grifolin	*Albatrellus confluens* & *Boletus pseudocalopus*	CNE1-LMP1 nasopharyngeal carcinoma cells	Targeted DNMT1 to block aerobic glycolysis. Blocked DNMT1 localization to restore OXPHOS	[112]
Quercetin	*Glycyrrhizia uralensis* or *G. glabra*	SNU719 gastric carcinoma cells	Induced cell cycle arrest and strong early apoptosis and necrosis/late apoptosis. Inhibited infection of EBV from lymphocytes to gastric adenocarcinoma cells.	[113]
Manassantin B	*Saururus chinensis*	P3HR-1 Burkitt lymphoma cells	Blocked lytic replication and virion production by inhibiting mTORC2 activity and blocking the mTORC2-PKC/AKT-signalling pathway.	[114]
Protoapigenone	*Thelypteris torresiana*	P3HR-1 Burkitt lymphoma cells	ZTA transactivation important for lytic cycle activation was inhibited.	[115]
Curcumin	*Curcuma longa*	HONE1 and HK1-EBV nasopharyngeal carcinoma cells	Induced cell cycle arrest and apoptosis via the mitochondria- and death receptor-mediated pathways.	[74]
		HeLa cervical cancer cells	Inhibited the transcription level of *EBNA1*.	[74]
Andrographolide	*Andrographis paniculata*	P3HR1 Burkitt lymphoma cells	Inhibited the transcription of *IE* genes that encoded Rta and Zta	[75]
Thirteen *ent*-rosane-type diterpenoids	*Euphorbia milii*	P3HR-1 Burkitt lymphoma cells	Inhibited lytic replication.	[87]
Twenty-six *neo*-clerodane diterpenoids	*Scutellaria barbata*	P3HR-1 Burkitt lymphoma cells	Inhibited lytic replication.	[86]
Moronic acid	*Rhus chinensis*	HEK, P3HR-1 Burkitt lymphoma cells	Inhibited the expression of Rta, and Zta, and interfered with the function of Rta.	[116]
28 lignans	*Saururus chinensis*	P3HR-1 Burkitt lymphoma cells	Inhibited lytic replication.	[117]
Polysaccharide	*Astragalus membranaceus*	Raji Burkitt lymphoma cells	Suppressed the expression of the IE protein, Rta, Zta, and EA-D.	[118]
Sulphated polysaccharides	Microalgae *Ankistrodesmus convolutus*, *Synechococcus elongatus*, and *Spirulina platensis*	Akata, B95-8, and P3HR- Burkitt lymphoma cells	Reduced cell-free EBV DNA.	[119]
Angelicin	*Psoralea corylifolia*	BC-3 and BCBL-1 lymphoma, and B95-8 and Raji Burkitt lymphoma cells	Inhibited lytic replication.	[120]
Lawsone (2-hydroxy-1,4-naphthoquinone)	*Lawsonia inermis* (henna leaf)	In vivo two-stage mouse skin model	Reduced EBV-early antigen activation Reduced skin carcinogenesis in a mouse model.	[121]
Luteolin (3,4,5,7-tetrahydroxyflavone)	Fruits and vegetables	NA, HA nasopharyngeal carcinoma, and P3HR-1 Burkitt lymphoma cells	Suppressed the activities of Zta and Rta by deregulating its Sp1 binding.	[122]

*Saururus chinensis* has been used as an anti-inflammatory, antipyretic agent to treat jaundice and edema in traditional Chinese and Korean medicine. Lignans, isolated from *S. chinensis* and *Litsea verticillate* exhibited an antiviral effect against EBV through inhibition of the lytic cycle along with other biological activity [117,123]. In particular, among 28 lignans isolated from *S. chinensis*, manassantin B showed the most promising inhibition [117].

The sulphated polysaccharides found in microalgae were also reported to have antiviral activity. For instance, the methanol extracts of *Synechococcus elongatus* and Ankistrodesmus convolutus were reported with low cytotoxicity and a strong antiviral effect against EBV in Burkitt’s lymphoma cells. The antiviral activity was measured by reducing the cell-free EBV DNA [119].

Moronic acid found in *Rhus chinensis* and Brazilian propolis inhibited the expression of Rta, Zta, and an EBV early protein [116]. It was also reported that moronic acid interfered with the function of Rta. In particular, it reduced the ability of Rta to activate a promoter containing a Rta-response element [116]. As the expression of many EBV lytic genes depends on Rta, the treatment of P3HR1 Burkitt’s lymphoma cells with moronic acid substantially reduced EBV particles produced by the lytic cycle [116].

*Astragalus membranaceus* has been used in traditional Chinese medicine for centuries primarily due to its immune-stimulatory, antiviral, antioxidant, and antitumor properties. The plant extract inhibited EBV lytic cycle by suppressing the expression of the immediate–early protein, including Zta, Rta, and EA-D [118]. The authors reported that the antiviral activities of *A. membranaceus* were attributed to its polysaccharides [118].

Henna (*Lawsonia inermis* L.) has been used to treat various ailments, including having antimalarial, antibacterial, and antidiabetic uses for centuries [121]. This plant is found in Africa, southern Asia, and part of Australia. Henna leaf powder and its primary pigment, lawsone (2-hydroxy-1,4-naphthoquinone), both showed significant inhibition (>88%) of EBV-early antigen activation in vitro. Additionally, henna leaf powder and lawsone significantly reduced skin carcinogenesis incidence in a mouse model administered by either oral or topical routes [121].

Cordyceps belong to the genus of Ascomycete fungi. Cordyceps are well known for their medicinal properties [124]. For instance, cordycepin, an adenosine derivative, found in cordyceps, expressed antitumor, antiviral and antifungal properties in previous studies [124]. Since cordycepin has a similar chemical structure to adenosine, it can be intercalated into RNA molecules and can terminate RNA synthesis [124]. Cordycepin can be either isolated from cordyceps or be produced synthetically and was reported to significantly downregulate most EBV genes of those tested [124]. The EBV genome copy number was reduced by up to 55%, in response to 125 µM cordycepin treatment. Furthermore, cordycepin significantly suppressed EBV transmission from cell to cell in a coculture [124]. Therefore, cordyceps should be further investigated for their activity against gamma herpesvirus infection and resulting cancers.

Manassantin B, a natural lignan extracted from the roots of the Asian lizard’s tail plant (*Saururus chinensis*), was found to be efficient in blocking EBV lytic replication and virion production with lower cytotoxicity [114]. The underlying mechanism by which manassantin B targets the viral lytic replication was through suppression of the *BZLF1* gene expression by interrupting the AP-1 signal transduction [114]. It specifically blocked the rapamycin complex 2 (mTORC2)-mediated phosphorylation of AKT Ser/Thr protein kinase at Ser-473 and protein kinase Cα (PKCα) at Ser-657. Briefly, Manassantin B inhibited mTORC2 activity by blocking the mTORC2-PKC/AKT-signalling pathway [114].

DNA methylation is an integral part of the EBV lytic cycle in which the viral genome is methylated during the latency period, and it gradually becomes unmethylated in the lytic cycles [125]. In EBV-associated NPC patients, abnormal DNA methylation has been found, which correlates with poor survival outcomes. Targeting this DNMT1, which causes abnormal DNA hypermethylation, is vital for epigenetic cancer therapy [125]. Natural compounds such as Grifolin have demonstrated DNMT1 expression inhibition and mitochondrial translocation [112].

### 4.1. Antiviral Agents from Food Sources

Recent research has also found effective EBV antiviral agents such as resveratrol and EGCG from regular foods such as vegetables and spices. Resveratrol is a natural phenolic compound found in many plants and fruits. The antiviral activity of resveratrol was reported for the influenza A virus, HIV, polyomavirus, and Herpesviridae family [126]. De Leo et al. demonstrated that resveratrol strongly induced apoptosis of EBV-positive Burkitt’s lymphoma cells depending on the viral latency program [97]. In particular, resveratrol greatly suppressed latency I EBV (+) cells, but not latency II and III cells (Figure 2). Additionally, resveratrol inhibited EBV reactivation by suppressing the lytic genes expression [96,97]. The production of virion was also reduced in a dose-dependent manner with the resveratrol treatment.

The popular green tea catechin EGCG is also a potential candidate for antiviral drug development. EGCG inhibited the expression of EBV lytic protein by blocking the transcription of immediate-early genes [102]. Liu et al. reported that EGCG could effectively inhibit the EBV lytic cycle at DNA, transcription, and protein levels [103,104]. Similarly, the dietary flavonoid apigenin, abundant in parsley, chamomile, celery, and vine-spinach, blocked the EBV lytic cycle by suppressing the expression of immediate–early lytic protein Zta, Rta, EA-D, and DNase and significantly reduced the production of EBV virion [99].

In comparison to EGCG and apigenin, luteolin (3,4,5,7-tetrahydroxyflavone) and protoapigenone inhibited EBV by suppressing the activity of immediate–early lytic protein [115,122]. Protoapigenone, a natural derivative of apigenin, inhibited the transactivation function of Zta and had no effect on the ability of Rta [115]. Additionally, protoapigenone was not toxic to the P3HR1 cells at the concentration that inhibit the function of Zta, indicating its therapeutic potential (selectivity index) in preventing EBV lytic proliferation [115]. Luteolin, a popular flavonoid has been reported to have several therapeutic properties including anti-inflammatory, antioxidant, antidiabetic, and antiangiogenesis activities. Luteolin also exhibited a significant inhibitory effect on EBV reactivation by suppressing Zta and Rta activities via deregulating its Sp1 binding, resulting in reduced virion production [122].

Angelicin is an angular furocoumarin naturally found in the seeds of *Psoralea corylifolia*, the roots of *Angelica archangelica*, and the family of Umbelliferae plants. Angelicin has been used to treat various skin diseases together with long-wavelength UV irradiation [120]. It efficiently inhibited lytic replication of murine gammaherpesvirus 68 (MHV-68) in a dose-dependent manner [120].

Ramayanti et al. reported that curcumin and its analogues significantly induced EBV reactivation in nasopharyngeal and gastric carcinomas cells [127]. Moreover, curcuminoids, especially compound EF24 (curcumin with the replacement of the β-diketone by piperidinone), displayed exceptional potential as an EBV lytic activator [127]. Furthermore, curcumin was found to reduce the viability and promote the apoptosis of EBV (+) nasopharyngeal carcinoma cells [127]. It also inhibited the proliferation of tumour cells by targeting the expression of EBV nuclear antigen 1 and exerted an antitumor effect [74].

### 4.2. Structural Modifications of Natural Products for Enhanced Activity

Natural antiviral agents thus far have not been highly effective in inhibiting EBV and have often displayed cytotoxicity, which limits their application to treat EBV infections. Structural modification of natural compounds and synthesis of new compounds have been conducted in the literature to overcome these limitations. For example, coumarin (+)-rutamarin, found in *Ruta graveolens*, moderately inhibited the EBV lytic cycle (IC_50_ = 7.0 µM) and its potency was not adequate for further investigation [100]. Twenty-eight (-)-rutamarin were semisynthesized from (-) chalepin, which is a derivative of (+)-rutamarin [100]. Of these, sixteen compounds were potent against EBV lytic DNA replication. The most effective antiviral compound had an IC_50_ value of 0.83 µM (dropped ninefold compared to unmodified (+)-rutamarin) and a selectivity index value of >120 [100]. 

Protoflavones are natural flavonoids, well known for antitumor properties [128]. However, their cytotoxicity against normal cells is a major concern [129]. Protoapigenone, a protoflavone, was reported to have an inhibitory effect on the EBV lytic cycle [129]. Twenty-seven compounds were prepared from apigenin, aiming to create protoflavones with less cytotoxic effect [129]. Among these semisynthesis derivatives, protoapigenone 1′-O-isopropyl ether was a promising lead with significant activity against EBV, low cytotoxicity, and a selectivity index of 73 [129].

GAP31 (gelonium anti-HIV protein, 31 kDa) protein, first isolated from the plant *Gelonium multifloru*, has shown great antiviral activities against HIV and HPV 1 infection [130]. The authors also reported the antiviral activities of GAP31 (synthetic) against EBV via targeting *EBNA1*, the only latent gene expressed in most EBV neoplasms [131]. *EBNA1* binds to the replication origin (oriP) to display its biological impact on EBV-driven cell transformation and maintenance [131]. Recombinant GAP31 blocked *EBNA1* dimer formation and consequently impaired EBNA1/oriP binding and transcription [131]. The blocking effect of GAP31 on *EBNA1*/oriP-dependent functions included impairing EBV-driven cell proliferation, transformation, and tumorigenesis [131]. In particular, rGAP31 at 0.32 nM reduced approximately 90% of the lymphoblastoid cell lines proliferation by day 15 in that study [131]. Furthermore, the EBV-driven cell transformation outgrowth was entirely abrogated by 0.64 nM rGAP31 [131]. GAP31 also suppressed EBV-mediated cancer formation and reduced the tumour size in the xenograft mouse model [131]. 

## 5. Conclusions and Future Direction

Natural sources with great chemical diversity could be the key to discovering novel, potent, and safe antiviral agents against EBV. However, the research on the therapeutic effect of gut microbial metabolites and natural products against EBV infection and cancers is limited. Natural bioactive compounds in plants, fruits and vegetables, microbes, fungi, and marine organisms have been shown to have significant antiviral and anticancer properties in the current literature. Most of the natural antiviral agents studied against EBV are phenolic compounds that suppressed expression and/or interfered with the functions of the EBV lytic proteins with some compounds inhibiting *EBNA1*. A few agents acted as nucleoside analogues and terminated RNA synthesis. Compounds such as triptolide, wogonin, and berberine have been tested in animal models for their efficacy against EBV-induced infection. Furthermore, the structural modification of natural compounds can be an effective method to improve their efficacy and overcome the limitations (such as cytotoxicity) of the parent compounds. Although several candidates have shown great potential for future drug development and their molecular mechanisms of action are understood up to some extent, further in vivo, and clinical studies are necessary. In particular, understanding their molecular mechanisms of action through more systematic in vitro and in vivo studies is crucial before conducting clinical trials. Similarly, future research is warranted to investigate the effectiveness and molecular mechanisms of gut microbial metabolites and gut microbiome in general against EBV-induced diseases at both preclinical and clinical levels. The potential role of probiotic foods against EBV could be another exciting area of research.

## Figures and Tables

**Table 1:** Potential antiviral effect and the proposed mechanism of action of short-chain fatty acids (SCFAs).

SCFA	Viral Infection	Type of Study	Description and Outcome of the Study	References
Acetate	Respiratory syncytial virus (RSV)	In vitro	Pretreatment of the A549 cells with acetate resulted in a reduction of RSV infection and increased expressions of retinoic acid-inducible gene-I (*RIG-I*) and interferon-stimulated gene15 (*ISG15*).	[54]
		In vivo	The intranasal treatment of acetate in mice reduced RSV infection and viral load and increased expression of *RIG-I* and Interferon β1 (IFNB1) in the lung tissue.	
		Ex vivo	Ex vivo treatment with acetate in RSV-positive infants showed reduced viral load and increased expression of *ISG15, RIG-I*, and MAVS (mitochondrial antiviral-signalling protein).	
		Clinical	Stools from 17 RSV-positive infants showed that the increased levels of acetate were associated with faster recovery, a shorter duration of fever, and higher oxygen saturation at admission.	
Propionate	Herpes simplex virus 1 (HSV-1)	In vivo	Mice were given sodium propionate supplement in drinking water three weeks before induction of the corneal HSV-1 infection, the animals had fewer ocular lesions when compared to the control group. Lymphoid and corneal tissues showed lower levels of CD4 (T-helper) Th1 and Th17 cells, macrophages, and neutrophils and higher levels of regulatory T cells (Treg) when compared to the control.	[55]
Butyrate	SARS-CoV-2 (COVID-19)	Clinical	High throughput RNA sequencing of many genes related to SARS-CoV-2 infection (4526 upregulated genes and 3167 downregulated genes) in butyrate-treated organoids and showed that butyrate downregulates genes essential for SARS-CoV-2 infection, such as *ACE2* and its associated genes. Butyrate was also found to upregulate toll-like receptors (TLR) and other antiviral pathways.	[56]

## Data Availability

No new data were created.
